# Effect of Sex on Motor Function, Lesion Size, and Neuropathic Pain after Contusion Spinal Cord Injury in Mice

**DOI:** 10.1089/neu.2019.6931

**Published:** 2020-08-26

**Authors:** Katelyn McFarlane, Taylor E. Otto, William M. Bailey, Amy K. Veldhorst, Renée R. Donahue, Bradley K. Taylor, John C. Gensel

**Affiliations:** ^1^ Spinal Cord and Brain Injury Research Center and Department of Physiology, University of Kentucky College of Medicine, Lexington, Kentucky, USA.; ^2^ Department of Anesthesia and Perioperative Medicine, Pittsburgh Center for Pain Research, and Pittsburgh Project to End Opioid Misuse, University of Pittsburgh School of Medicine, Pittsburgh, Pennsylvania, USA.

**Keywords:** biological variable, brain, gender, hypersensitivity

## Abstract

Spinal cord injury (SCI) causes neurodegeneration, impairs locomotor function, and impacts the quality of life particularly in those individuals in whom neuropathic pain develops. Whether the time course of neurodegeneration, locomotor impairment, or neuropathic pain varies with sex, however, remains understudied. Therefore, the objective of this study in male and female C57BL/6 mice was to evaluate the following outcomes for six weeks after a 75-kdyn thoracic contusion SCI: locomotor function using the Basso Mouse Scale (BMS); spinal cord tissue sparing and rostral-caudal lesion length; and mechanical allodynia and heat hyperalgesia using hindpaw application of Von Frey filaments or radiant heat stimuli, respectively. Although motor function was largely similar between sexes, all of the males, but only half of the females, recovered plantar stepping. Rostral-caudal lesion length was shorter in females than in males. Mechanical allodynia and heat hyperalgesia after SCI developed in all animals, regardless of sex; there were no differences in pain outcomes between sexes. We conclude that contusion SCI yields subtle sex differences in mice depending on the outcome measure but no significant differences in behavioral signs of neuropathic pain.

## Introduction

Spinal cord injury (SCI) is a devastating disability that historically has disproportionally affected young men. The demographics of SCI are changing, however, with increased incidence among older individuals and women.^1^ In response to National Institutes of Health (NIH) initiatives to investigate sex as a biological variable in pre-clinical animal studies,^2^ there is increased focus on sex differences in recovery from neurotrauma^3,4^ leading to an emerging literature on sexual dimorphisms in central nervous system (CNS) injury. After thoracic (T8) contusion SCI in rats, females have significantly improved locomotor and anatomical recovery relative to males.^5^ Whether these sex differences in motor function and neuropathology extend to mice is not well understood.

The incidence of chronic neuropathic pain after SCI is 65–80%.^6^ After SCI, women present a higher prevalence of nociceptive pain and consume greater amounts of analgesic drugs including opioids and nonsteroidal anti-inflammatory drugs (NSAIDs).^7^ By contrast, after SCI in rats, mechanical hypersensitivity occurs with greater frequency in males compared with females.^8,9^ The extent to which sex differences in central neuropathic pain occur in SCI mice is understudied. Here we explored the role of sex in functional and anatomical recovery and the development of pain in mice after contusive SCI.

## Methods

### Animals

Twenty-four female and 24 male, four-month old C57BL/6 mice were used to generate the data for this study (Jackson Laboratory, Bar Harbor, ME). Animals were housed in individually ventilated cages with *ad libitum* access to food and water. Housing is set to maintain a 14 h light/10 hr night cycle at 70°F and 50% humidity. All experimental procedures were conducted during the light cycle and with the approval of the Institutional Animal Care and Use Committee at the University of Kentucky.

### Surgery

Animals were anesthetized with intraperitoneal injections of ketamine (100 mg/kg) and xylazine (10 mg/kg). There were six experimental groups: (1) male with 75 kdyne SCI, *n* = 10; (2) female with 75 kdyne SCI, *n* = 10; (3) male sham (laminectomy only), *n* = 8; (4) female sham, n = 8; (5) male no injury (anesthetic only), *n* = 6; and (6) female no injury *n* = 6. The hair overlying the thoracic region of the spinal cord was shaved for all animals.

For Groups 1–4 (SCI and sham), the skin was incised, and the connective and muscle tissue was dissected to expose the vertebral column from T8–T10. A dorsal laminectomy was performed at T9. Animals were then placed under the Infinite Horizons (IH) injury device (Precision Systems and Instrumentation).^10^ Groups 1–2 received a moderate-severe contusion SCI (75 kdyn displacement). Spinal cord displacement at the time of SCI was the same for both sexes (female 594 ± 20 mm; male 580 ± 24 mm; *p* = 0.65) ensuring that similar injury severities were achieved in males and females. Groups 3-4 were removed from the injury device without receiving SCI.

Muscle and skin incisions were then closed using monofilament sutures. Immediately after surgery, all mice were given one subcutaneous injection of buprenorphine-SR (1 mg/kg) and one subcutaneous injection of an antibiotic agent (5 mg/kg, enrofloxacin 2.27%: Norbook Inc, Lenexa, KS, dissolved in 2 mL saline). Animals recovered on paper towels in cages on ∼37^0^C heating pad overnight before returning to home cages. Subcutaneous antibiotic injections (5 mg/kg, enrofloxacin) were given in 1 mL saline for five days. Manual bladder expression was performed on injured mice twice daily until sacrificed. Four SCI animals died because of post-surgical complications: three in Group 1 (male-SCI) and one in Group 2 (female-SCI).

### Tissue processing

Mice were anesthetized and transcardially perfused with cold phosphate buffered saline (PBS) (0.1 M, pH 7.4: Na_2_HPO_4_ (Fisher, Cat#7558-79-4), NaH_2_PO_4_ (Fisher, 10049-21-5), NaCl (Fisher, 7210-16)), followed by perfusion with cold 4% paraformaldehyde (PFA, Alfa Aesar, 30525-89-4) at 42 days post-injury (dpi). One cm of the spinal cord centered on the laminectomy site (groups 1–4) or on T9 was dissected from each animal and then post-fixed in 4% PFA for 2 h and subsequently rinsed and stored overnight in phosphate buffer (PB, 0.2 M, pH 7.4) at 4°C.

The following day, tissues were cryoprotected in 30% sucrose for 3–5 days at 4°C, followed by rapid freezing and blocking in optimal cutting temperature (OCT) compound (Sakura Finetek USA, Inc.) on dry ice. Spinal cords from Groups 1–4 were distributed randomly (by an experimenter blinded to group assignment) to tissue blocks to ensure every group was represented on each slide. The blocked tissue was stored at −80°C before sectioning. Transverse serial sections (10 μm) were cut through each block and mounted on Colorfrost Plus Slides (Fisher, 12-550-17), and then stored at −80°C before staining.

### Immunohistochemistry and quantification

Slides were placed on a slide warmer set to 37°C to facilitate adherence of spinal cord sections. Slides were then rinsed with 0.1-M PBS and incubated in blocking buffer (0.1-M PBS with 1% bovine serum albumin (Fisher, BP1605), 0.1% Triton X-100 (Sigma, X-100), and 5% normal goat serum (Sigma, G9203)) at room temperature for 1 h, followed by incubation in blocking buffer containing primary antibody overnight at 4°C. Primary antibodies were chicken anti-Neurofilament (NF)(1:1000, Aves, Cat #NFH) and chicken anti-glial fibrillary acidic protein (GFAP) (1:500, Aves, Cat # B00094).

On the second day, slides were rinsed in 0.1-M PBS and then incubated with the secondary antibody, biotinylated goat anti-chicken (1:500, Aves, Cat#B1005) at room temperature for 1 h. Slides were rinsed with 0.1-M PBS, then incubated with Elite-ABC (Vector PK-6100) for 1 h at room temperature and then incubated with 3,3'-Diaminobenzidine or SG (Vector SK-4100; SK-4700). Slides were rinsed and then stained with either fast-red (FR) counterstain (Vector Labs, H-3403) or eriochrome (solochrome) cyanine (EC)(Sigma, E2502) for myelin.

Slides were dehydrated in a series of ethanol (70%, 95%, 100%, VWR, 71002-508, 86125-164, EM-EX0276-4S) and histoclear (VWR, 101412-878), then mounted with coverslips in Permount (Fisher, SP15-500). All antibodies were previously tested with a dilution curve (ranging from 1:100-1:5000), including a nonprimary control, to confirm optimal staining concentrations.

We selected tissue sections with lesions (containing 5% or more of unmyelinated/demyelinated areas) for quantification of spared and total spinal cord cross-sectional tissue areas using GFAP/FR staining. All images were captured with a ZEISS Axio Scan.Z1 slide scanner with a 20x objective. Previously, we established that GFAP expression coincides with areas of spared axons at chronic time points after mouse SCI.^11^ To quantify spared tissue area, the regions of dense GFAP-positive staining were outlined and measured using the Halo Image Analysis software (Inca Labs, New Mexico). The percentage of spared tissue at the lesion epicenter (i.e., section with least amount of spared tissue) was calculated by dividing the spared tissue area by the total cross-sectional area of the spinal cord.

The EC/NF stains were used to estimate the rostral-caudal extent of the lesion—i.e., the lesion length. We estimated the lesion length as the distance between the most rostral and the most caudal tissue sections containing lesion. Two animals, one male SCI (Group 1) and one female SCI (Group 2) were excluded from anatomical analyses because of extensive tissue loss during processing.

### Behavioral analyses of locomotor function

Locomotor recovery was assessed using the Basso Mouse Scale (BMS) at 1, 3, 7, 14, 21, 28, and 42 dpi as reported previously.^12,13^ Each hindlimb was scored separately based on hindlimb movement (e.g., ankle placement and stepping), coordination, and trunk stability. For statistical analysis, both limbs were averaged to generate a single score for each animal. A score of zero indicates complete paralysis while a score of nine indicates normal locomotion.

Plantar stepping ability was derived from the BMS.^12^ As described previously,^12^ each hindlimb was scored as having none, occasional, frequent, or consistent plantar stepping. This depended on whether weight support and plantar placement (along with swing and stance) were maintained while the animal was moving for less the 50% of the time (occasional), greater than 50% of the time but not all the time (frequent), or all of the time (consistent). Each hindlimb was then scored as either none, occasional, frequent, or consistently plantar stepping.

### Behavioral analyses of sensory function

Behavioral indices of neuropathic pain were measured before surgery and repeated at two, four, and six weeks after surgery. Mechanical sensitivity was assessed with the up-down method using von Frey (Vf) monofilaments.^14^ Animals were first acclimated to the testing apparatus consisting of a wire mesh floor within an acrylic enclosure. A monofilament was pressed perpendicularly against the plantar surface of the hindlimb until bent, beginning with the 1.4 gf monofilaments and ranging from the 0.4 gf to 6.0g f monofilaments. A 50% withdrawal threshold was calculated for each animal and reported as the average of both hindpaws at each time point.^15^

Heat sensitivity, also known as the Hargreaves test,^16^ was assessed as previously described.^17^ Animals were first acclimated to the testing apparatus (Ugo Basile, Germany), consisting of a glass floor within an acrylic enclosure, for 1 h. An infrared laser beam located under the floor and set to an infrared intensity of 25 units was aimed at the plantar skin surface of the hindlimb and activated. The latency to a unilateral hindpaw withdrawal response (e.g., jumping, licking, and flicking) was automatically recorded. A cutoff time of 25 sec was used to avoid tissue injury. A total of five trials per limb were taken for each animal at each time point. Testing was alternated between limbs with an intertest duration of at least 5 min. An average across the five recorded trials and across hindlimbs was computed for each animal at each time point.

### Statistical analyses

All data acquisition and analyses were performed by investigators blinded to experimental conditions. Statistical analyses were completed using GraphPad Prism 8.0 (GraphPad Software). Planned comparisons utilizing two-way repeated measures analysis of variance followed by Bonferroni *post hoc* tests were used to evaluate locomotor and pain outcomes. Specifically, the effects of Sex × Time (repeated) on BMS scores were analyzed for SCI animals. Individual, planned comparisons between male and female SCI groups, male and female sham groups, and within sex sham versus SCI groups were used to evaluate the effect of Sex and Injury on pain outcome measures; Time was treated as a repeated measure for all comparisons.

For sex comparisons of SCI-specific outcomes (SCI displacement, lesion length, and tissue sparing measures) unpaired *t* tests were used to compare the male and female groups. To analyze stepping frequency, a chi-square test was performed. The sham (laminectomy alone) and no injury (anesthetic alone) groups were not different in behavior (BMS score 9) or on any pain outcomes (*p* > 0.05, main effect of surgery) and therefore only sham (laminectomy alone) groups were used for statistical analyses. Simple linear regression was used to correlate injury and anatomical outcomes with functional results. Results were considered statistically significant at *p* < 0.05.

## Results

**FIG. 1.**
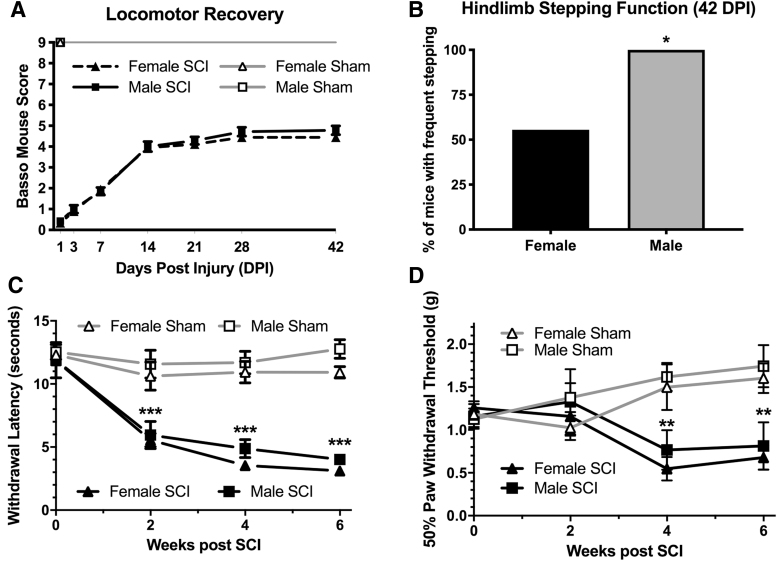
Sex alters locomotor recovery but not pain responses after spinal cord injury (SCI). Adult (4-month-old) male and female mice received a moderate-severe thoracic contusion SCI (75 kdyn Infinite Horizons T9 contusion). (**A**) Locomotor recovery (Basso Mouse Scale, BMS) was the same for both male and female mice (main effect of sex *p* = 0.5) with both groups achieving plantar stepping by 42 days post-injury (dpi). (**B**) Males had significantly improved plantar stepping frequencies compared with females at 42 dpi (*chi-square, *p* = 0.04). Locomotor function for shams of both sexes was normal (BMS = 9) by one day after SCI. (**C,D**) Both thermal (heat) and mechanical (von Frey) hypersensitivities developed in male and female mice after SCI. Thermal responses differed significantly between sex-matched sham and SCI starting two weeks post-injury (***p < 0.001) and four weeks for mechanical responses (**p < 0.01), Bonferroni *post hoc* tests after repeated measures analysis of variance. There were no differences between sexes. *n* = 6/sham; *n* = 7–9/SCI per sex; mean ± standard error of the mean.

As illustrated in Figure 1A, SCI decreased BMS scores of gross locomotor function in both male and female mice. The BMS scores did not differ between SCI males and females over the six-week observation period (F 1,14 = 0.52, *p* = 0.48 main effect of sex). The average BMS for both sexes was between 4 and 5 at 42 dpi. To better understand the effect of sex on locomotor recovery, we examined individual locomotor parameters observed with BMS scores of 4–5 (i.e., plantar stepping frequencies and coordination).

As illustrated in Figure 1B, further segregation of plantar stepping abilities revealed a significant improvement in plantar stepping frequency in males: 100% of male SCI mice recovered frequent stepping (plantar stepping during the majority of locomotor activity) in at least one hindlimb by 42 dpi (7/7), while only 55% (5/9) of females SCI had frequent stepping of at least one hindlimb at this time point (chi-square, *p* = 0.04). One male (1/7) recovered some forelimb-hindlimb coordination while no females (0/9) recovered coordination.

To test the hypothesis that the time course of central neuropathic pain in a mouse SCI contusion model is sex-dependent, we examined plantar withdrawal responses to heat (infrared) and mechanical (Von Frey) stimuli. As illustrated in Figure 1C,1D, baseline measurements for withdrawal responses to heat and mechanical stimuli were similar across groups (*p* > 0.5).

As illustrated in Figure 1C, when collapsed across both sexes, SCI decreased heat withdrawal latency throughout the six-week testing period (F3, 90 = 25.9, *p* < 0.0001, Time × Injury interaction). Within each sex, SCI significantly decreased withdrawal latency to heat in male SCI versus male shams (F1, 13 = 45.1, *p* < 0.0001, main effect of SCI) and female SCI vs. female shams (F1, 15 = 160, *p* < 0.001) at 2-, 4-, and 6-weeks post-injury (*p* < 0.001, Fig. 1C). Between sexes, however, withdrawal latency to heat did not differ over time between male and female shams (F1, 14 = 2.4, *p* = 0.15, main effect of sex; F3, 42 = 0.7, *p* = 0.77 Sex × Time interaction) or male and female SCI (F1, 14 = 1.1, *p* = 0.30 main effect of sex; F3, 42 = 0.48, *p* = 0.70 Sex × Time interaction).

As illustrated in Figure 1D, when collapsed across both sexes, SCI decreased mechanical thresholds through the six-week testing period (F3,90 = 9.0, *p* < 0.0001, Time × Injury interaction). Within each sex, SCI significantly decreased mechanical thresholds in males (SCI vs. shams; F1, 13 = 6.2, *p* = 0.027, main effect of injury) and females (SCI vs. shams; F1, 15 = 19.3, *p* = 0.0005, main effect of injury) at four and six weeks post-injury (*p* < 0.05, Fig. 1D).

Mechanical thresholds, however, did not differ over time between sexes for male versus female shams (F1, 14 = 1.5, *p* = 0.24, main effect of sex; F3, 14 = 0.43, *p* = 0.73 Sex × Time interaction) or male versus female SCI (F1, 14 = 0.43, *p* = 0.52, main effect of sex; F3, 42 = 0.25, *p* = 0.86 sex × time interaction). Collectively, these data indicate that SCI produces hypersensitivity in both males and females to the same degree and time course.

**FIG. 2.**
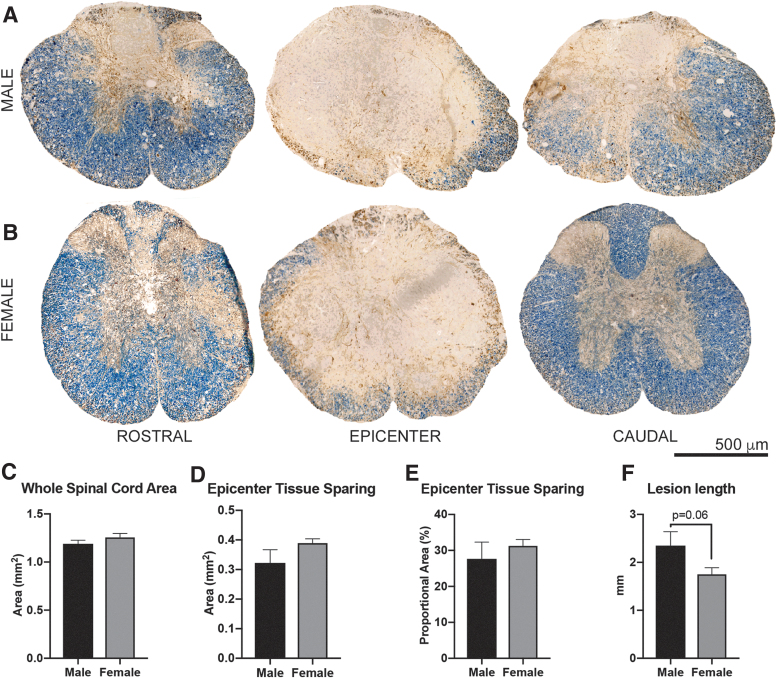
Anatomical changes after spinal cord injury (SCI) between male and female mice. Adult (4-month-old) male and female mice received a moderate-severe thoracic contusion SCI (75 kdyn Infinite Horizons T9 contusion), and then tissue was collected at 42 days post-SCI. (**A**) Representative T9 spinal cord tissue sections from male (**A**) and female (**B**) SCI mice spanning 1.6 mm rostral-caudal centered on the lesion epicenter: EC (blue-myelin) and NF (axons-brown). (**C–E**) There were no significant differences between sexes in spinal cord size (*p* = 0.26), epicenter spared tissue area (*p* = 0.18), or proportional area of spared tissue (*p* = 0.49) at 42 days post-injury (*t* tests). (**F**) Female mice have a shorter lesion length compared with males (*p* = 0.06, *t* test) which is also evident in rostral and caudal tissue sections in (A). Mean ± standard error of the mean, *n* = 6–8 per sex. EC, eriochrome cyanine; NF, neurofilament. Color image is available online.

Next, we evaluated the effect of sex on spinal cord pathology at 42 dpi. As evident in Figure 2A, contusion injuries produced areas of extensive tissue damage with a small rim of spared tissue at the lesion epicenter. The lesion spanned roughly 2 mm of the spinal cord along the rostral-caudal axis. At the lesion epicenter, there were no significant differences in tissue sparing in females versus males in overall cross-sectional area (females = 1.26 ± 0.04 mm^2^, males = 1.19 ± 0.04 mm^2^; *p* = 0.26), spared tissue area (females = 0.39 ± 0.01 mm^2^, males = 0.32 ± 0.04 mm^2^; *p* = 0.18), and the percentage of spared tissue (females = 31.2 ± 1.8%, males = 28.8 ± 3.3%; *p* = 0.20) at the lesion epicenter (Fig. 2D,2E). The rostral-caudal lesion length was marginally reduced in females (1.75 ± 0.14 mm) compared with males (2.35 ± 0.29 mm), but this missed statistical significance (*p* = 0.065, Fig. 2F).

**FIG. 3.**
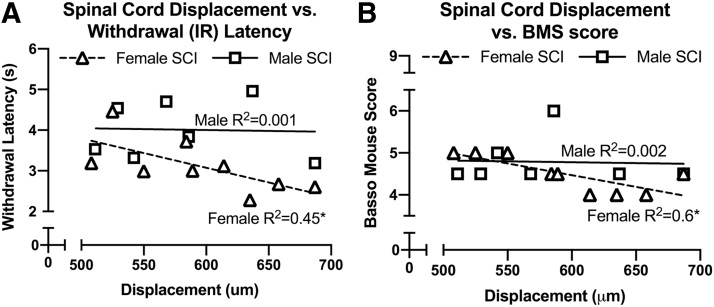
Injury parameters correlate with locomotor and pain responses in female, but not male, spinal cord injury (SCI) mice. Spinal cord displacement values were recorded at the time of contusion injury (75 kdyn Infinite Horizons T9 contusion) in adult (4-month-old) male and female mice and correlated to functional outcomes at end-point (42 days post-SCI). (**A,B**) Linear regression analyses show that inverse correlations exist between displacement and open field locomotor performance (Basso Mouse Scale [BMS], **B**), as well as withdrawal latency to an infrared (IR) heat stimulus (**A**). Correlations were significant (*p < 0.05) for female SCI mice but not for male SCI mice. The coefficient of determination (R-squared) is included in each respective graph. Additional regression analyses are in Table 1.

We next examined the correlation between functional tests (BMS as well as heat and mechanical withdrawal responses) and measures of injury severity (spinal cord tissue displacement at the time of SCI, lesion length, and tissue sparing). As illustrated in Figure 3 and Table 1, BMS was significantly correlated with tissue displacement. Also, withdrawal responses to heat were significantly correlated with tissue displacement. In both cases, this was true only for females and not males. There were no significant correlations between other functional tests and measures of injury severity for males or females (Table 1).

**Table 1.** Linear Correlational Analyses^*^

BMS, Basso Mouse Scale; IR, infrared; Vf, von Frey.

^*^ All outcomes from 42 days post-injury except tissue displacement (determined at time of SCI from the Infinite Horizons injury device). Sparing measures calculated at the lesion epicenter as in Figure 2.

## Discussion

The NIH initiatives to support the investigation of sex as a biological variable in pre-clinical animal studies^2^ bring to bear the importance of understanding the extent to which males and females recover from neurotrauma and other neuropathologies.^3,4^ In the current study, we observed that male mice exhibit improvements, albeit modest, in recovery of motor function compared with females after thoracic contusion SCI. In contrast, SCI did not significantly change pathological tissue damage; if anything, we observed a slight, although not significant (*p* = 0.065), increase in tissue preservation in females relative to males. Tissue displacement at the time of injury significantly correlated with functional outcomes (BMS and heat hypersensitivity) in female but not male SCI mice. Interestingly, for both mechanical allodynia and thermal (heat) hyperalgesia measures, the time course of development and magnitude of chronic impairment was similar between male and females after SCI.

Over a six-week period after thoracic SCI, we observed that all male mice, but only just over half of female mice, recovered hindlimb plantar stepping. Our results of increased stepping function in males are inconsistent with previous studies of SCI in mice and rats.

First, after a T10 compression SCI in mice, females recovered hindlimb plantar stepping and some forelimb-hindlimb coordination, while males were limited to hindlimb weight support with inconsistent plantar stepping.^18^ Second, after T12 compression SCI in mice, females recovered hindlimb weight support while males recovered only hindlimb joint movement.^19^ Third, after T8 contusion SCI in rats, females recovered plantar stepping and coordination while males recovered only hindlimb stepping without coordination.^5^ Fourth, in the same rat model, gait parameters such as hindlimb swing, stance, and support were significantly improved in females compared with males.^5,20^ Fifth, again in the T8 contusion model in rats, females recovered hindlimb plantar placement, a prerequisite for stepping, while males only recovered hindlimb movement.^19^

Thus, in previous reports, females consistently demonstrate improved stepping function relative to males across multiple species and models of SCI. One possible explanation for our differing result could be related to our use of buprenorphine as a post-operative analgesic, or the administration of antibiotic agents after SCI. Another possible explanation could be related to our observation of increased mortality/morbidity in males (three) versus females (one) after SCI—a selection bias that might favor males with improved recovery and thus plantar stepping.

We observed that SCI produces a slightly smaller lesion size in females compared with males. The neuroprotective effects were not large in that we did not detect sex differences in tissue sparing. Pronounced neuroprotection in females across multiple outcomes (lesion length, lesion volume, tissue sparing) was not observed here or in previous studies. Specifically, Farooque and colleagues^18^ reported the qualitative observation that, 14 days after compression SCI in mice, spinal cord damage was more extensive in males compared with females.

Second, in rats, Swartz and coworkers^21^ detected shorter lesion lengths and increased tissue sparing in females compared with males when measured one month after T9 contusion SCI. Third, in rats, Datto and associates^5^ reported reduced lesion volume and increased tissue sparing in females when assessed at 13 weeks after T9 contusion SCI; however, the overall rostral-caudal lesion length was comparable for both sexes. Collectively, the spinal cords of rats and mice after SCI exhibit less signs of damage in females compared with males, although the effects are not robust across studies nor across outcome measures.

We observed similar, subtle sexual dimorphisms when we examined correlations between injury severity (tissue displacement produced by the impactor at the time of SCI) and behavioral outcomes. Specifically, tissue displacement was correlated with locomotor recovery and with withdrawal responses to heat stimuli in female, but not male, SCI mice. This is consistent with a report in rats that detected stronger correlations between tissue sparing measures and locomotor recovery (Basso, Beattie, Bresnahan [BBB] score^22^) in females compared with males.^5^

Increased attrition, resulting in a smaller sample size, may have reduced our ability to detect significant correlations in male SCI mice in the current study. It is worth noting, however, that the IH impactor, injury device used to produce SCI in mice, was developed and optimized using females while the NYU/MASCIC injury device used to produce rat SCI was developed using both sexes. Specifically, the injury parameters for rats with the NYU/MASCIS SCI impactor used by Datto and colleagues,^5^ were optimized to correlate with functional (BBB) and lesion outcomes in both male and female rats after SCI.^23^ In contrast, the injury parameters for the IH impactor were optimized using only females^10,24^ and the BMS was also developed using only female SCI mice.^12^ Thorough examination of sex as a biological variable in mouse SCI studies may require further refinement of injury and behavioral assessment techniques to accommodate sex-specific subtleties in recovery.

This type of refinement may be required for insight into the specific mechanisms, such as sex hormones, that underlie sexual dimorphisms in SCI. Indeed, evidence from hormone treatment studies in SCI support the rationale that estrogen or progesterone affects SCI recovery (reviewed by ^4,25^). Interestingly, most treatment studies are performed using a single sex in isolation.

The few studies that examine the role of sex hormones in both sexes, by comparing intact males and females with females receiving ovariectomy before SCI, implicate female sex hormones as advantageous for recovery. After cervical hemisection SCI, female rats had improved respiratory function compared with males with the advantage lost in ovariectomized females.^26^ Similarly, at-level allodynia developed in fewer female rats compared with males after thoracic contusion SCI; however, the proportions were normalized between sexes when females received an ovariectomy before injury.^9^ Both of these studies implicate sex hormones in sexual dimorphisms observed in SCI recovery.

We observed that SCI produced comparable heat hypersensitivity and mechanical allodynia in male and female mice. Our findings contrast with a recent SCI study in rats by Gaudet and coworkers.^8^ They reported that contusion produced mechanical allodynia in males but not females. Other than a possible species difference, one source of inconsistency is that we produced contusion injury without using residual compression, while Gaudet and coworkers^8^ utilized a 1 sec sustained compression.^8^

We observed previously that residual compression after contusion SCI potentiates pro-inflammatory macrophage activation.^13^ Because inflammation is associated with the development of pain after SCI,^27,28^ it is possible that compression contributes to macrophage activation in a sex-dependent manner. Whether these types of injury biomechanics induce sexually dimorphic inflammatory responses is an interesting question for future studies.

Pain symptoms after SCI are associated with changes within the spinal cord at the level of injury as well as distal to the site of injury in both the lumbar spinal cord and the brain.^27,29–31^ Stimulus-evoked withdrawal responses can engage reflex circuits and may be indicative of increased spasticity in addition to neuropathic pain. In the current study, however, most withdrawal responses were accompanied by shaking or licking of the paw, thought to represent supraspinal processing of pain.

More complex behavioral analyses are warranted, including those that engage the affective components of pain, to provide for a more thorough mechanistic insight into sexual dimorphisms.^32^ Indeed, SCI pain likely results from pathology to a combination not only of the sensory discriminative and affective components of pain, but also organizational and activational systems. It is well established that sex-specific organizational changes, those that occur during development and during periods of sexual maturation, result in structural differences in the nervous system between sexes.^33^ Organizational differences influence behavior and functional responses. In addition, activational, or transient, sex-specific hormonal changes in response to SCI also likely affect pain outcomes.

As mentioned above, SCI pain responses are reduced with ovariectomy.^9^ The SCI also induces a robust inflammatory response at the site of injury and distal regions of the CNS that are implicated in the development and maintenance of chronic pain.^27,28,34^ Interestingly, sexual dimorphisms in inflammation contribute to pain responses after peripheral injury,^35^ and we determined previously that immunomodulatory drugs exert sex-specific analgesic effects after SCI.^17^

## Conclusion

Our results demonstrate that contusion SCI yields subtle sex differences in mice depending on the outcome measure with both sexes developing mechanical allodynia and thermal (heat) hyperalgesia in response to injury. The majority of NIH-funded rodent SCI research is conducted exclusively in females^36^; however, the significance of sex as a biological variable in SCI disease progression may vary with SCI biomechanics or for different functional and therapeutic outcomes. Indeed, we recently reported sexually dimorphic responses to SCI analgesic therapies despite similarities in the development of pain between males and females.^17^ Collectively, our observations here along with those reported in previous studies highlight considering sex as a biological variable in SCI research.
